# Total and Free Zinc Dynamics as Biomarkers for Neurological Impairment in Traumatic Spinal Cord Injury

**DOI:** 10.3390/nu17030496

**Published:** 2025-01-29

**Authors:** Raban Arved Heller, Maria Maares, Thilo Samson Chillon, Hanno Witte, Obada T. Al-Halabi, Stefan Heene, Alexander Younsi, Patrick Haubruck, Lutz Schomburg, Arash Moghaddam, Bahram Biglari, Hajo Haase

**Affiliations:** 1 Institute for Experimental Endocrinology, Charité—Universitätsmedizin Berlin, Corporate Member of Freie, Universität Berlin, Humboldt-Universität zu Berlin, Berlin Institute of Health, 13353 Berlin, Germany; raban.heller@charite.de (R.A.H.); thilo.chillon@charite.de (T.S.C.); lutz.schomburg@charite.de (L.S.); 2 Department of Food Chemistry and Toxicology, Technische Universität Berlin, Straße des 17. Juni 135, 10623 Berlin, Germany; maares@tu-berlin.de; 3 Department of Traumatology and Orthopaedics, Septic and Reconstructive Surgery, Bundeswehr Hospital Berlin, 10115 Berlin, Germany; 4 Department of Hematology and Oncology, Bundeswehr Hospital Ulm, 89081 Ulm, Germany; hanno.witte@uni-ulm.de; 5 Department of Neurosurgery, Heidelberg University Hospital, Im Neuenheimer Feld 400, 69120 Heidelberg, Germany; obada.alhalabi@med.uni-heidelberg.de (O.T.A.-H.); alexander.younsi@med.uni-heidelberg.de (A.Y.); 6 Medical Faculty, Heidelberg University, Grabengasse 1, 69117 Heidelberg, Germany; 7 Raymond-Purves Bone and Joint Research Laboratories, Kolling Institute of Medical Research, Institute of Bone and Joint Research, University of Sydney, St Leonards, NSW 2065, Australia; patrick.haubruck@sydney.edu.au; 8 Orthopedic and Trauma Surgery, Frohsinnstraße 12, 63739 Aschaffenburg, Germany; 9 Department of Paraplegiology, BG Trauma Centre Ludwigshafen, 67071 Ludwigshafen, Germany; bahram.biglari@bgu-ludwigshafen.de

**Keywords:** traumatic spinal cord injury (TSCI), zinc (Zn), free zinc (fZn), biomarker signature, neurological impairment, oxidative stress, inflammation, diagnostic

## Abstract

*Background/Objectives:* Traumatic spinal cord injury (TSCI) profoundly impacts patients by precipitating a loss of motor and sensory capabilities, largely due to oxidative stress and inflammation during the secondary injury phase. *Methods:* This investigation explores the diagnostic potential of zinc (Zn) and free zinc (fZn) as biomarkers by analyzing their serum concentration dynamics in 48 TSCI individuals with TSCI, with the aim of correlating these levels with neurological impairment. Serum samples collected at admission, 4 h, 9 h, 12 h, 24 h, and 3 days post-injury were analyzed for total serum Zn and fZn concentrations. The patients were compared to a control group comprised of individuals with vertebral fractures but no neurological deficits. *Results:* The study revealed injury-specific fluctuations in Zn and fZn levels following TSCI, with significantly lower Zn levels observed post-TSCI compared to controls (*p* = 0.016). The American Spinal Injury Association (ASIA) Impairment scale (AIS) assessments at admission and three months post-injury showed Zn level differences are linked to neurological recovery (AIS+:1 > AIS+:2, 0 h: *p* = 0.008; AIS+:0 < AIS+:1, 4 h: *p* = 0.016), highlighting the critical role of Zn and trace elements in the early remission process after TSCI. Notably, significant differences in fZn levels were detected between the control and TSCI groups (TSCI < Control; 12 h: *p* = 0.045; 24 h: *p* = 0.001; 3 d: *p* = 0.016), with the peak diagnostic performance of fZn at 24 h post-injury, as indicated by an Area Under the ROC Curve (AUC) of 83.84% (CI: 0.698–0.978). *Conclusions:* These findings underscore the potential of fZn as a biomarker to guide early diagnostic and therapeutic interventions aimed at mitigating secondary injury and enhancing recovery outcomes. This study contributes insights into the dynamics of serum Zn and its importance, holding specific diagnostic properties that could be critically relevant in the early phase of biomarker signature development for TSCI diagnostics and prognosis.

## 1. Introduction

Traumatic spinal cord injury (TSCI) is a severe condition in traumatology, primarily affecting young individuals and leading to substantial physical, psychosocial, and economic impacts [1]. The pathophysiology of TSCI unfolds in phases, starting with immediate mechanical damage in the primary phase, followed by a secondary phase characterized by complex inflammatory responses and autonomic dysregulations [2]. Despite advancements in early surgical interventions post-TSCI [3], the challenge remains in promptly and accurately diagnosing and treating patients, especially when they are unable to comply with clinical testing due to factors such as coma or sedation.

Zinc (Zn), an essential trace element, plays a crucial role in various biological processes [4,5], including those related to SCI [6]. It is involved in antioxidant defense, immune function, and neural signaling [5,7,8]. The body maintains Zn balance through absorption and homeostatic mechanisms, with Zn existing in two primary forms: tightly and labile bound Zn, the latter also being known as free zinc (fZn) [5]. fZn plays a crucial role in severe health conditions such as sepsis owing to its dynamic properties and participation in cellular signaling and inflammatory processes [5,9].

Post-SCI, patients experience considerable stress and pronounced inflammatory responses, factors that have the potential to disrupt zinc metabolism [7,10,11]. Zn is one of the basic immune response modulators [4,5,7,10,12]—a feature of utmost significance in SCI—since inflammation is a major component of this disease [6,11]. The anti-oxidative role of Zn is significant in the battle against oxidative stress, the predominant characteristic after SCI [4,7,13]. In this context, changes in zinc dynamics, particularly in serum free zinc levels, serve as indicators of the body’s physiological and pathological cellular responses [14]. Studies have shown great diagnostic value for fZn in its ability to reflect the immune status [8,15].

The role of Zn in neural health, neuroprotection, and neurodegeneration has been relatively well explored in TSCI [6,11,16,17,18]. Furthermore, synaptic transmission and plasticity underscore the potential of this element to affect the recovery after SCI [19,20]. On the whole, the Zn status is an important component of nutritional status in the general scheme of overall health and recovery in humans. In this regard, dietary interventions, supplementation, and chelator treatment strategies share the potential to have an effect on the status of Zn and neurological health outcomes [19,20,21,22,23,24].

With the influence of Zn at a basic regulatory level of the immune system and the involvement of the metal during an early immune response [4,7], it stands as a possible biomarker in the evaluation of neurological damage post-TSCI [11,18]. Deviations in Zn concentration in the serum, especially during the first 24 h of inflammation induced by a trauma, would be regarded as characteristic of biological response to injury [11,18,25,26]. Additionally, the role of Zn in maintaining the integrity of neurons and in neuronal damage and repair processes in other conditions, including traumatic brain injury and stroke, suggests the importance of Zn in TSCI [19,20].

Recent studies suggest that measuring total Zn levels in serum post-TSCI offers valuable diagnostic and prognostic insights [6,11,18]. Thus, the ratio of total Zn to fZn in serum could offer more nuanced insights into the severity of the injury and the body’s homeostasis of inflammatory response. This ratio may be particularly relevant in assessing the degree of neurological impairment, providing a finer granularity of information that is closely linked to clinically meaningful endpoints [9,18]. This ratio, as well as the dynamics of fZn, might provide valuable diagnostic tools for the estimation of the risk of neurological impairment, odds for neurological recovery, monitoring therapy, and help in identifying further therapeutic options [6,27].

In animal and human studies, research further revealed the importance of fZn in immune function and inflammation [9,28,29]. Knowledge about the mechanisms of action of Zn on immune function and inflammatory response allows for precisely targeted intervention not only in the field of immunology but also in traumatology [30,31,32,33]. The aim of this study is to observe the changes in Zn homeostasis and the alterations in fZn and its ratio to total Zn in the context of TSCI. We are, therefore, interested in characterizing these dynamics in relation to clinical covariates such as the NLI (neurological level of injury), which might give us insights into the implications that the Zn status could have on the recovery process and general health outcomes in patients with TSCI.

The study of Zn levels and fZn homeostasis in human serum after a TSCI provides a new way to understand the complex biochemical and immune responses that occur following such injuries. We hypothesize that Zn homeostasis and the regulation of fZn can serve as part of a diagnostic and prognostic signature after TSCI. By linking Zn homeostasis characteristics with clinical factors and neurological outcomes, we aim to gain a better understanding of TSCI and how to manage it effectively. This could lead to improved patient care and rehabilitation strategies. This research is essential for improving clinical management and designing targeted nutraceutical and therapeutic interventions to facilitate recovery following TSCI.

## 2. Materials and Methods

This study obtained approval from the ethics committee of Rheinland Pfalz (Number: 837.266.09) 5 October 2009, the University of Heidelberg’s local Ethics Committee (S514/2011) 7 February 2012 and was registered at the German Clinical Trial Register (Deutsches Register Klinischer Studien—DRKS|Study-ID: DRKS00009917|Date of Registration: 23 March 2016|Universal Trial Number (UTN): U1111-1179-1620). Data collection and processing adhered to good scientific and clinical practice, and all participants provided written informed consent. They were informed of their right to withdraw from the study without negative consequences. The study was performed following the principles of the Declaration of Helsinki [34], and adheres to the TRIPOD statement (Transparent Reporting of a multivariable prediction model for Individual Prognosis or Diagnosis) [35].

### 2.1. Data Source

**Figure 1:**
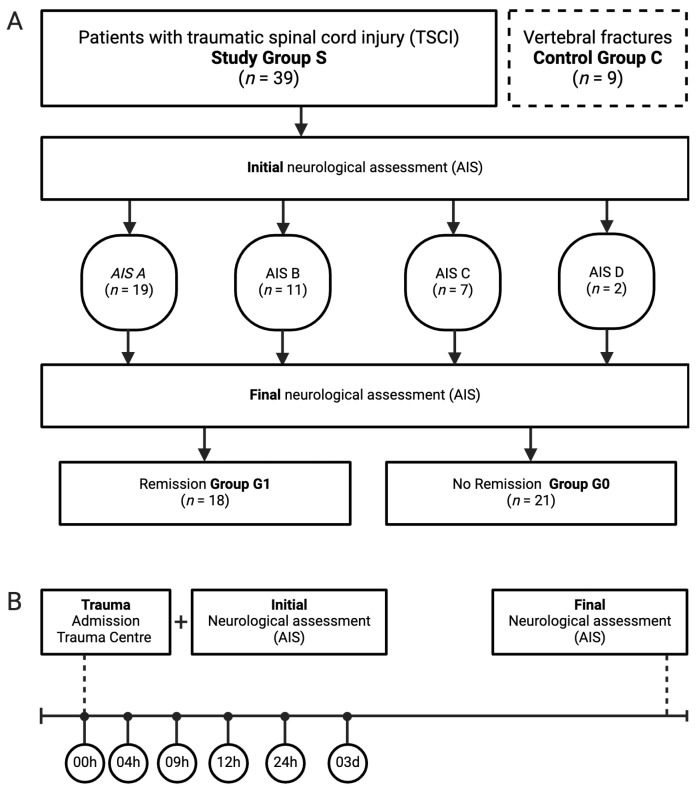
Protocol for patient and material collection. (**A**) Prospective enrollment of patients with TSCI was conducted; individuals with vertebral fractures but no neurological deficits were included as a control group. (**B**) A standardized blood sampling protocol was implemented for each enrolled patient. Beginning at admission, six blood samples were collected at the following times: immediately (0 h), 4 h, 9 h, 12 h, 1 day, and 3 days post-TSCI. Neurological evaluations were carried out upon admission and again three months later.

Patient data were obtained from the hospital database. Admission time may not correspond directly to the moment of injury, as delays in hospital arrival can affect the timing of initial blood samples. The time to admission did not exceed 4 h [36]. Venous blood samples (7.5 mL Monovette, Sarstedt, Germany) were collected post-injury at specific time points over 72 h from admission to follow-up, as outlined in our protocol (Figure 1B). Following 20 min of coagulation, the samples were centrifuged at 3000 rpm and stored at −80 °C for subsequent analysis as previously detailed [11]. Clinical covariates, which encompass the NLI (characterized as the most inferior level with preserved motor and sensory capabilities), were documented from the hospital’s database, alongside the AIS, as depicted in Figure 1A.

#### 2.1.1. Quantification of Total Serum Zn

The study was carried out in an S1 laboratory at Charité Universitätsmedizin Berlin. Zn levels were analyzed using total reflection X-ray fluorescence (TXRF) technique. The inter-assay coefficients of variation (CV) were typically less than 10%. The total serum Zn concentration was measured with a benchtop TXRF spectrometer (S4 T-STAR, Bruker Nano GmbH, Berlin, Germany) [37]. Briefly, samples were diluted 1:2 (*v*/*v*) with a gallium standard (1000 μg/L). Then, 8 μL of the diluted solution was applied to a polished quartz glass slide and allowed to dry overnight. The fluorescence generated from X-ray activation was captured by the benchtop TXRF spectrometer to determine the trace element concentrations from the emission spectrum.

#### 2.1.2. fZn Measurement and Ratio of Free to Total Zn

The free Zn concentration was measured in an S1 laboratory at Technische Universität Berlin using a fluorimetric technique with the Zinpyr-1 sensor, a low-molecular-weight Zn sensor from Santa Cruz Biotechnology in Dallas, TX, USA, as outlined in previous studies [8,27]. In brief, 20 μL of serum sample, pre-diluted in a 1:10 ratio with assay buffer and stored at −80 °C, was mixed with 80 μL of prewarmed assay buffer containing Zinpyr-1 (final concentration 0.05 μM ). The sensor’s saturation level was determined by adding 15 μL of either EDTA (800 μM) or ZnSO_4_ (4.5 mM) to elicit minimal and maximal fluorescence signals of Zinpyr-1, respectively. Free Zn concentrations in serum were calculated based on the dissociation constant (Kd) for the Zinpyr-1-Zn complex, which is 0.7 nM. The ratio of free Zn to total Zn per sample was determined using the following equation:

$${\mathrm{Zn}}_{\mathrm{ratio}}=\left(\frac{\mathrm{fZn}\;\left[\mathrm{nmol}/L\right]\times 65.38\;\left[g/\mathrm{mol}\right]}{\mathrm{Total}\;\mathrm{Zn}\;[\mu g/L]}\right)\times 10^{2}$$

### 2.2. Participants

Venous blood samples were collected from traumatic spinal cord-injured patients between 2011 and 2017 at the Department of Paraplegiology, BG Trauma Center Ludwigshafen. All patients had at least one spinal fracture and were classified according to the AO classification [38]. Exclusion criteria included non-traumatic SCI, traumatic brain injury, severe abdominal trauma, traumatic amputation of extremities, coma or other life-threatening trauma apart from SCI. Methylprednisolone sodium succinate was not administered. Patients were assigned to the study group S (n = 39), consisting of two subgroups: G1 (n = 18) with neurological remission and G0 (n = 21) without neurological remission. A control group C (n = 9) comprised nine subjects with vertebral fractures without neurological impairment (Figure 1A).

### 2.3. Outcome

Neurological impairment was classified using AIS grades based on the International Standards for Neurological Classification of SCI (ISNCSCI). Initial examinations (AIS initial) were conducted within 72 h after admission, with final examinations (AIS final) at 3 months post-trauma. Neurological remission was defined as an improvement in AIS grade within 3 months.

### 2.4. Predictors

Individual Zn and fZn concentration patterns were analyzed concerning the presence or absence of neurological impairment and neurological remission.

### 2.5. Missing Data

Mean follow-up within the first 72 h was 77.4%, and missing values were excluded from the analysis via pairwise deletion.

### 2.6. Related Work

The registry was formerly utilized to study biomarkers for SCI. The analyses were conducted retrospectively while the samples were gathered prospectively. Consequently, varying patient numbers were included in particular analyses. Zn data had been previously published in part [11], and are included in this analysis to offer a thorough context for interpreting fZn dynamics.

### 2.7. Statistical Analysis

Non-parametric test methods were used to investigate location shifts between groups. Categorical variables were assessed using Boschloo’s exact test [39]. Multiple testing adjustments were not applied, p-values are to be interpreted descriptively. Univariate logistic regression evaluated predictive potential. Receiver Operator Characteristic (ROC) analysis determined the area under the curve (AUC) for predictive performance. Statistical calculations were performed using R version 4.3.2 [40], and figures were created using the package *ggplot2* [41].

## 3. Results

### 3.1. Demographics

We conducted a study with 48 patients, including 39 with traumatic spinal cord injuries and 9 with spine fractures without neurological impairment acting as controls. The cohort included 37 males and 11 females, followed for a duration of three months. The median age of the patient cohort was 53 years, with an interquartile range (IQR) ranging from 36 to 67 years, covering an age range from 20 to 85 years. Notably, there were no significant differences between the two groups, G0 and G1, in age, sex, AO-classification, and NLI. Comprehensive demographic data are displayed in Table 1 and Figure 1. Information on control group is available in Table 2.

**Figure 2:**
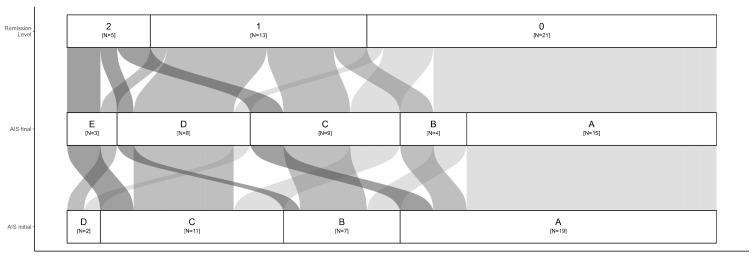
Sankey diagram illustrating AIS grade progression from initial assessment (AIS initial) to final assessment (AIS final) and remission levels. The width of the alluvial flows represents the number of patients transitioning between AIS grades over time. The stratum boxes are labeled with AIS grades and the number of patients (N) at each stage. Remission levels (0, 1, 2) indicate the patient transitions between AIS grades over the course of the study. AIS+0 represents no improvement, while AIS+1 and AIS+2 reflect one- and two-step improvements in neurological function.

The data highlight that a significant portion of patients initially classified as AIS A or B remained in lower remission categories (AIS+0), whereas patients with initial AIS C or D showed a higher likelihood of progressing to higher remission levels (AIS+1 or AIS+2). This suggests that baseline severity relates to recovery potential. The flow between AIS initial and AIS final demonstrates the variability in natural recovery trajectories. Notably, the highest remission levels (AIS+2) predominantly occurred in patients with less severe initial injuries (AIS C or D) (Figure 2).

### 3.2. Correlation of fZn to Total Zn

**Figure 3:**
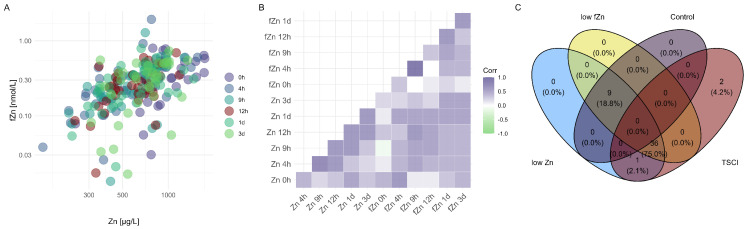
Correlation Analysis of Zn and fZn in TSCI and control groups over time. (Panel (**A**)) features a scatterplot of total Zn versus fZn concentrations, color-coded by days post-injury to illustrate temporal changes and correlation patterns within the TSCI and control cohorts. (Panel (**B**)) presents a correlation heatmap (corrplot) showing the strength and direction of the relationship between total Zn and fZn across all time points. (Panel (**C**)) includes a Venn diagram comparing groups with low Zn and low fZn levels among individuals with TSCI and controls, highlighting the overlap and distinct subsets within these categories. This comprehensive visualization aids in understanding the dynamics and potential diagnostic relevance of Zn and fZn in traumatic spinal cord injury.

To investigate the relationship between fZn and total Zn concentrations, correlation analyses were conducted for each day post-injury and by analyte (Figure 3A). The results indicated a significant overall correlation (r = 0.451, *p* < 0.001, $r^{2}$ = 0.2), with time-specific subpatterns observed for each analyte (Figure 3B). These correlation patterns suggest that changes in the total Zn levels may influence the availability of fZn, potentially affecting cellular signaling and immune responses. A majority of patients with spinal cord injury exhibited consistently lower concentrations of both Zn and fZn over time (Figure 3C).

### 3.3. Examination of the Complete Patient Group: Trauma Versus Control

**Figure 4:**
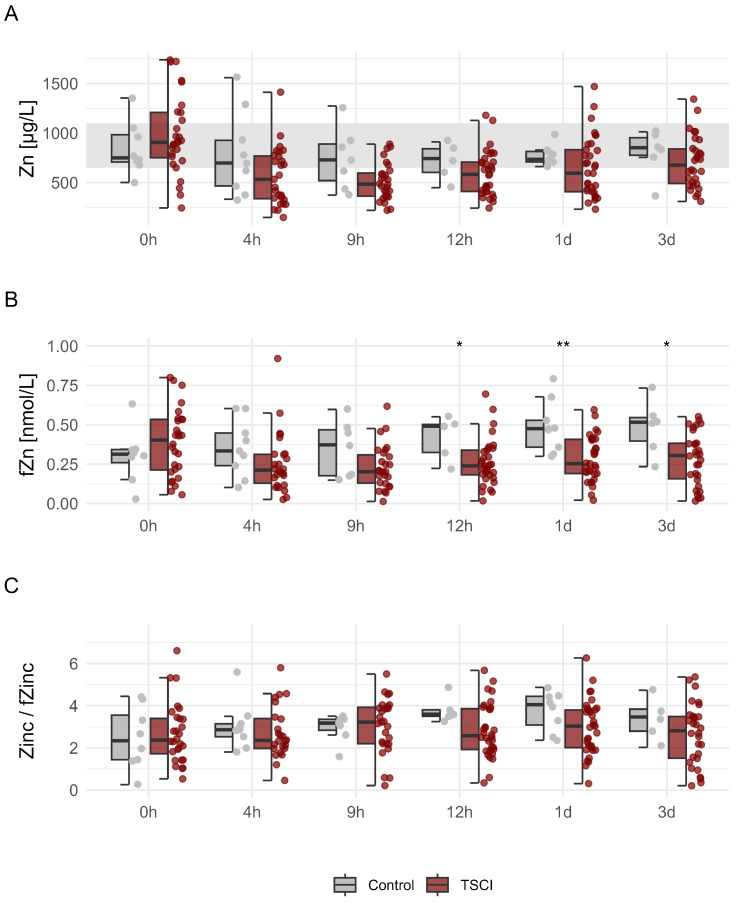
Dynamics of Serum Zn and fZn in individuals with TSCI compared to controls. Concentrations of total Zn (Panel (**A**)) and fZn (Panel (**B**)), as well as the ratio of total Zn to fZn (Panel (**C**)), measured at multiple time points post-TSCI. Panel A depicts the normal range of Zn (650–1100 μg/L) using gray shading to place serum Zn concentrations within appropriate limits. This figure contrasts the control group, which exhibits no neurological impairment, with individuals who sustained TSCI, highlighting significant temporal fluctuations in Zn metrics that potentially serve as diagnostic markers in the context of traumatic spinal cord injury. Differences between groups are marked for statistical significance utilizing the Mann–Whitney U test: * indicates p<0.05, and ** indicates p<0.01.

In line with our previous work [11], in the TSCI group our analysis showed a decrease in mean serum Zn levels within the first 4 h post-injury from 979 μg/L to 565 μg/L (Wilcoxon signed rank test, *p* = 0.0001). The lowest concentration occurred at 4 h with 149 μg/L after trauma in the TSCI group, with 56% of values falling below the physiological range of 650–1100 μg/L. Nine hours post-injury, 79% of Zn levels were measured below 650 μg/L. Subsequently, a gradual and increase in mean serum Zn levels was observed (Figure 4A) from 609 μg/L at 12 h to 689 μg/L after 3 d. At admission, the TSCI group exhibited higher mean serum fZn levels compared to controls, with a marked decrease observed up to 4 h post-injury. In contrast, the control group showed a trend to increase in fZn levels from 0 to 4 h post-admission, with the difference between the groups becoming significant up to 24 h after the injury (TSCI < Control; *p* = 0.0013) (Figure 4B). In contrast to the distinct patterns observed in serum Zn and fZn levels, the ratio of Zn to fZn did not exhibit a significant trend when comparing spinal cord injury patients to controls. This indicates that the ratio of Zn to fZn may not provide additional diagnostic value in distinguishing between these groups post-injury (Figure 4C).

### 3.4. Comparison of Total Zn and fZn Concentrations in Relation to Neurological Remission

**Figure 5:**
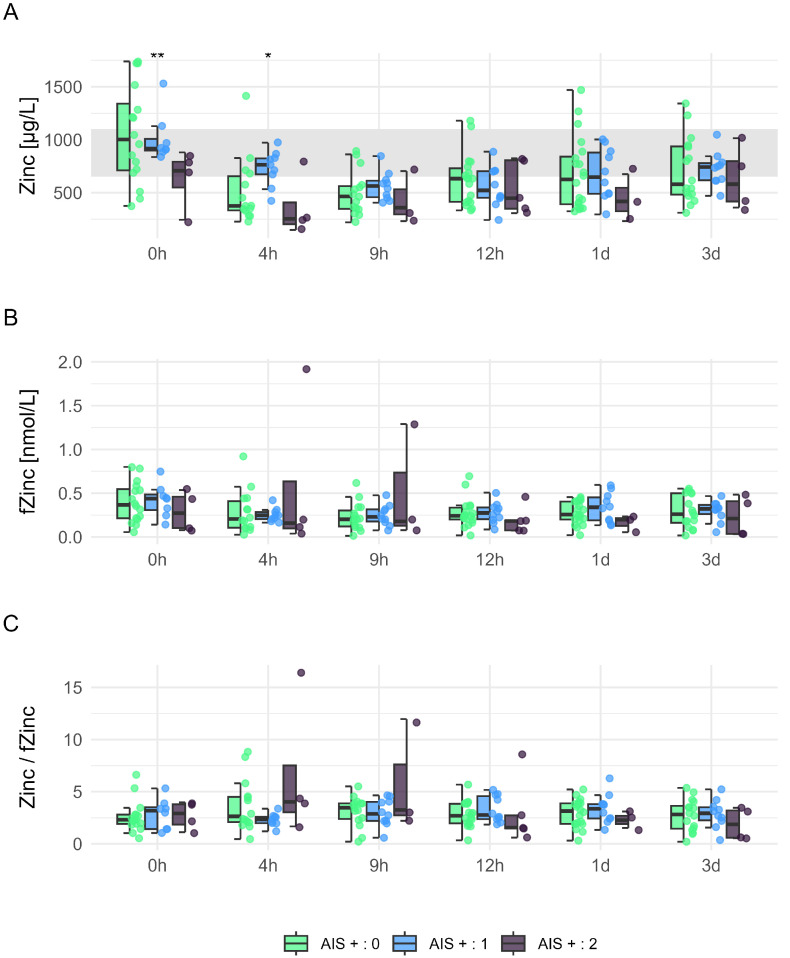
Dynamics of serum Zn and fZn in individuals who sustained TSCI by AIS conversion group. Concentrations of total Zn (Panel (**A**)) and fZn (Panel (**B**)), as well as the ratio of total Zn to fZn (Panel (**C**)), measured at multiple time points post-TSCI. Panel A showcases the standard physiological range of Zn (650–1100 μg/L), which is indicated with gray shading to facilitate evaluation of Zn levels within normal limits. This figure displays comparisons among individuals with TSCI classified into AIS conversion groups: AIS+0, AIS+1, and AIS+2, highlighting significant differences in Zn dynamics across varying degrees of neurological improvement. Differences between groups are marked for statistical significance utilizing the Mann–Whitney U test: * indicates p<0.05, and ** indicates p<0.01.

The data revealed a high variance in Zn dynamics, with significant differences observed at the first measurement point (AIS+:1 > AIS+:2, 0 h: *p* = 0.008) and at 4 h post-injury (AIS+:0 < AIS+:1, 4 h: *p* = 0.016) (Figure 5A). Additionally, high variances in fZn concentrations were specifically noted at 4 h and 9 h post-injury in patients with outstanding neurological recovery, indicating a potential fluctuation in fZn during the acute phase of spinal cord injury (Figure 5B). Despite these variances in Zn and fZn levels, no significant differences were found in the Zn to fZn ratio across different grades of neurological remission. These findings suggest that while total serum Zn may correlate with initial neurological outcomes, the variability in fZn concentrations and their ratios do not consistently align with specific neurological recovery profiles (Figure 5B,C).

### 3.5. Serum and fZn Dynamics in TSCI Patients Classified by NLI and Initial Injury Severity

**Figure 6:**
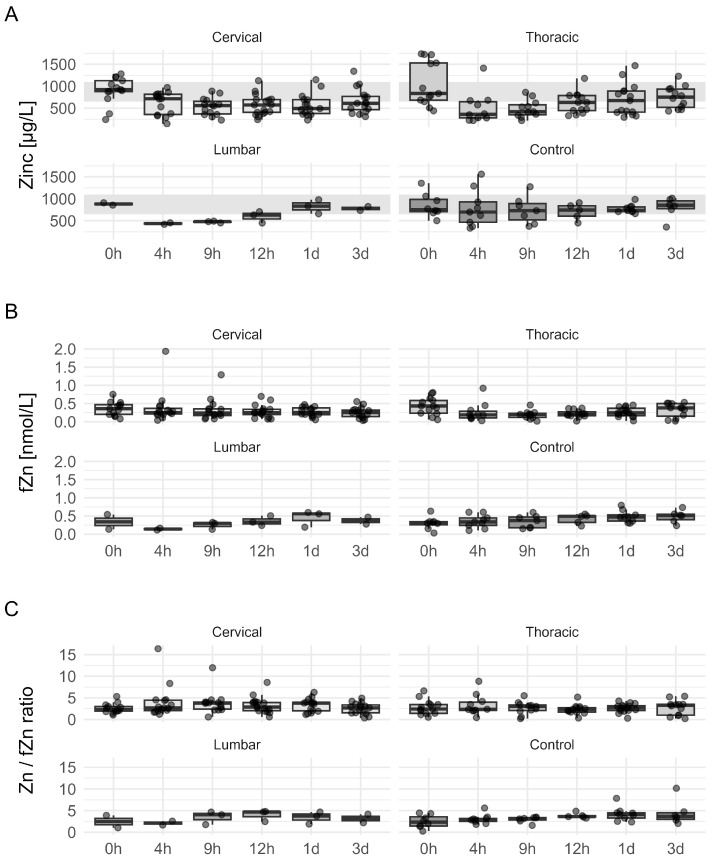
Dynamics of serum Zn and fZn in TSCI patients categorized by neurological injury level and control. Concentrations of total Zn (Panel (**A**)) and fZn (Panel (**B**)), as well as the ratio of total Zn to fZn (Panel (**C**)), measured at multiple time points post-TSCI. Each panel is faceted to show data grouped by neurological injury level, along with a control group. Panel A illustrates the normal physiological range of Zn (650–1100 μg/L) with gray shading to provide context for serum Zn concentrations within typical limits.

Investigating the data, categorized by their NLI distinct pattern in the decrease in serum Zn (Zn) levels after the trauma was most pronounced in patients who sustained thoracic injuries. This suggests that the extent of Zn depletion may be linked to both the severity and location of the spinal injury (Figure 6A). In contrast, when examining the variance of fZn and the Zn to fZn ratio across different NLI subgroups, the results indicated a comparably low variance and difference between NLI groups over time. This stability suggests that while total serum Zn levels fluctuate significantly following injury, the fZn ratios to total Zn remain relatively consistent across different levels of neurological injury. This pattern of Zn dynamics may provide insights into the systemic and localized responses following TSCI, highlighting the potential role of Zn monitoring in patient management and prognosis (Figure 6B,C).

**Figure 7:**
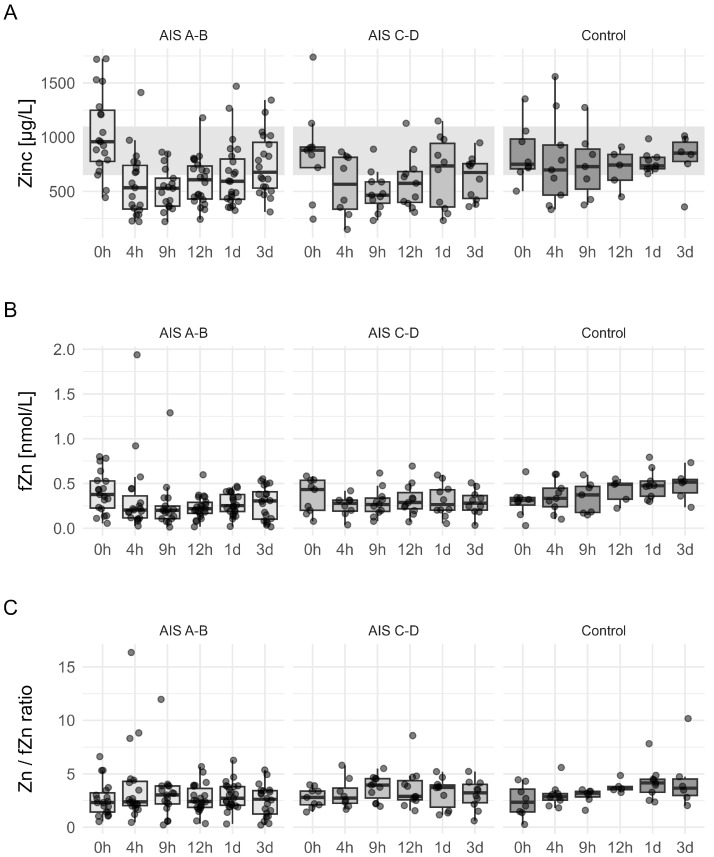
Box plots display the trends of Zn distribution, separated by initial injury severity (AIS A-B, AIS C-D) and control groups, over time following traumatic spinal cord injury (TSCI). Total serum Zn levels [μg/L] were assessed at various intervals (0 h, 4 h, 9 h, 12 h, 1 d, 3 d), with the shaded region indicating the normal Zn range (650–1100 μg/L) (Panel (**A**)). fZn levels [nmol/L] were measured at the same intervals, showing reduced fZn levels in AIS A-B patients compared to AIS C-D and control groups (Panel (**B**)). The Zn/fZn ratio appeared mostly constant across different groups, with slight variations over time (Panel (**C**)).

Dynamics of Zn concentrations stratified by initial injury severity (AIS A-B, AIS C-D) and control groups revealed more insights into the relationship between injury severity and Zn homeostasis (Figure 7). Total serum Zn levels [μg/L] demonstrate a marked decrease in patients classified as AIS A-B compared to AIS C-D and control groups, with the most relevant mean reduction observed during the hyperacute phase (0 h to 12 h) and prominent deviation from normal levels in severely injured patients. fZn concentrations [nmol/L] revealed lower levels in AIS A-B patients at critical time points (12 h and 1 d) relative to less severe injury groups. Despite significant fluctuations in total Zn and fZn, panel C indicates that the Zn/fZn ratio remains stable across groups. This stratified analysis reinforces the link between greater initial injury severity and disturbances in Zn homeostasis, providing valuable insights into how Zn levels may predict neurological outcomes and recovery trajectories.

### 3.6. Overall Comparison of Zn and fZn in Relation to Impairment

**Figure 8:**
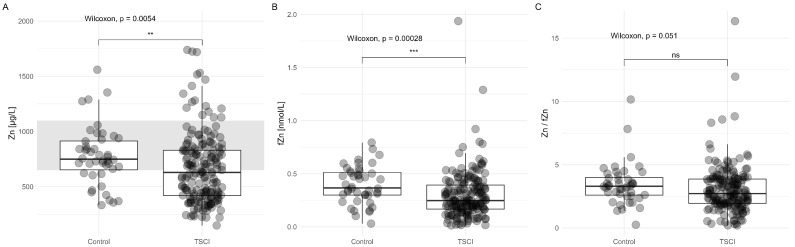
Global comparison of Serum Zn and fZn between TSCI patients and controls. (Panel (**A**)) features boxplots representing the total Zn levels in serum, aggregated over all time points, with a gray region denoting the normal physiological range of Zn (650–1100 μg/L) for reference. (Panel (**B**)) illustrates boxplots of fZn levels, pooled from all collected data. (Panel (**C**)) presents boxplots of the ratio of total Zn to fZn. Significant differences between TSCI patients and control groups are highlighted: ns denotes p>0.05, ** denotes p<0.01, and *** denotes p<0.001.

Our study identified notable differences between TSCI patients and control groups in total Zn levels (TSCI < Control; *p* = 0.0054), with an even greater disparity observed in fZn (TSCI < Control; *p* = 0.00028). These findings indicate that fZn levels are particularly affected in the TSCI group compared to controls. Additionally, the mean Zn levels in the TSCI group were below the physiological normal range (650–1100 μg/L), suggesting a substantial disruption of Zn homeostasis following spinal cord injury. Differences in the Zn to free Zn ratio were not significant (TSCI < Control; *p* = 0.051) (Figure 8).

### 3.7. Performance Analysis of Diagnostic Modeling Based on Zn and Free Zn Metrics

**Figure 9:**
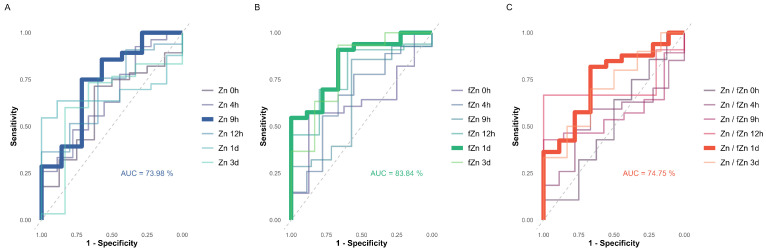
Univariate model ROC curves for predicting neurological impairment using Zn metrics. Each panel presents ROC curves calculated at various time points post-injury to evaluate the predictive power of Zn metrics for the detection of neurological impairment. (Panel (**A**)) features ROC curves based on total Zn concentrations, with the time point yielding the highest Area Under Curve (AUC) highlighted in a bold line. (Panel (**B**)) displays ROC curves for fZn, again with the most predictive time point emphasized in bold. (Panel (**C**)) illustrates the ROC curves for the ratio of total Zn to fZn, with the highest AUC time point distinctly marked. The AUC values are provided in the lower right corner of each panel, indicating the effectiveness of each metric at different times post-injury in predicting neurological outcomes. This visual representation underscores the temporal dynamics and diagnostic potential of Zn measurements in assessing TSCI.

Serum fZn concentrations in the TSCI group exhibited an increasing difference over time reaching the maximum 24 h after the injury (TSCI < Control; *p* = 0.0013) (Figure 4B). To evaluate the diagnostic value of serum Zn and fZn metrics for identifying patients at high risk of neurological impairment, univariate binary logistic regression analysis was performed per points in time and analyte to differentiate between TSCI patients and controls Figure 8. All models were contrasted based on AUC, with a table outlining the attributes of those models achieving the top AUC for each analyte across points in time Table 3. Model 1 reveals a significant negative association between Zn levels at 9 h post-injury and the outcome variable, indicating that decreased Zn concentrations at this early time point are linked to higher values of the outcome. Model 2 demonstrates a similar negative relationship for fZn levels at 1 day post-injury, suggesting that lower concentrations of fZn at this time point are associated with higher odds of neurological impairment. Similarly, Model 3 highlights a significant negative association between the ratio of Zn to fZn levels at 1 day post-injury and the outcome variable, indicating that a decrease in this ratio corresponds to higher values of the outcome. Effect sizes, as measured by Cohen’s d, indicate large effects for all predictors. The overall highest area under the curve (AUC) of 83.84% (CI: 0.6984–0.9783) was recorded for fZn levels 24 h after injury, with a substantial effect size (Cohen’s d) of 1.33. This finding suggests that fZn concentrations at 24 h post-injury may serve as a robust and early indicator of neurological impairment (Figure 9).

## 4. Discussion

This research examined in detail the alterations in Zn and fZn concentrations following TSCI, offering perspectives on their potential roles as biomarkers for neurological injury severity. We observed specific trends in the behavior of Zn and free Zn, notably marked within the initial 24 h post-injury. These differences suggest that utilizing Zn assessments along with other established markers as part of a panel could be valuable for the early detection and assessing the severity and prognosis of TSCI.

### 4.1. Zn Dynamics and Unbound Zn in Relation to Neurological Dysfunction

The significant decrease in total Zn levels during the first 9 h post-injury aligns with other research showing a notable depletion of trace metals after acute injuries [37]. This reduction in Zn levels post-TSCI may result from its increased use in several physiological processes that are upregulated due to the injury, including inflammation, immune response, and oxidative stress management [10,18]. The decrease was most pronounced in individuals with thoracic injuries, indicating that the level of injury, and injury severity may influence Zn depletion [11]. This supports the hypothesis that more severe injuries disrupt a broader range of bodily functions, including mineral metabolism [4,7,22].

The lack of significant differences in the Zn to fZn ratio prompts questions regarding whether homeostatic mechanisms that regulate Zn availability might compensate after traumatic spinal cord injury. The possibility exists that the precise regulation of fZn, despite variations in total Zn levels, aids in maintaining cell functions crucial for injury responses. This non-significant difference may also imply that both forms of Zn are similarly influenced by the injury and subsequent physiological changes, suggesting that the body might prioritize balancing bound and unbound Zn to facilitate recovery. These findings underscore the potential value of measuring both total and fZn levels as biomarkers for evaluating neurological impairment and overall physiological disruption following traumatic spinal cord injury (Figure 8).

### 4.2. Diagnostic Value of fZn Measures

Zn is essential for neuroprotection, synaptic function, and neurotransmission, making it crucial in trauma cases where nervous system integrity is at risk [16,19,20,23].

The correlation between Zn levels and the degree of inflammation is well-documented, with Zn being inversely related to inflammatory markers. Recent evidence on biomarkers studied alongside Zn include C-reactive protein (CRP), and cytokines confirmed a negative correlation between Zn levels and CRP, indicating that lower Zn levels are associated with higher inflammation [42,43,44].

Our results showed significant differences in the absolute concentrations of both Zn and fZn, while the ratio of Zn to free Zn did not show significant differences. This suggests that although the total amount of Zn decreases, the proportion available as free Zn does not change uniformly, indicating complex regulation of Zn availability post-TSCI that is not fully captured by the ratio alone. Notably, in the specific analysis using fZn levels at 24 h post-injury, the AUC was 83.84%, highlighting its potential as a robust biomarker for neurological impairment. Additionally, the changes in fZn at 4 and 9 h post-injury likely reflect metabolic disturbances and compensatory mechanisms triggered by the injury [4,7], which can vary greatly among patients depending on the severity of the injury and individual physiological responses [2,11,18].

Integrating Zn into a multi-marker signature could provide a more comprehensive assessment of inflammatory status and disease progress. Zn’s role in immune modulation often proves superior to other biomarkers, given its direct impact on both innate and adaptive immune responses. However, the idea of Zn supplementation should be critically examined. It is hypothesized that Zn deficiency might be a protective physiological response to mitigate excessive inflammation. Therefore, supplementation must be carefully considered to avoid disrupting this potential protective mechanism.

### 4.3. Molecular Mechanisms and Therapeutic Potential of fZn Dynamics in TSCI

The dynamic alterations in serum Zn concentrations observed in our study align with previous research [11,18,37] and are supported by findings from animal models [9]. In addition to mechanical trauma, metabolic processes in the secondary injury phase following TSCI can lead to cell death [45], particularly in astrocytes, oligodendrocytes, and neurons [16,45]. Glutamate excitotoxicity is a well-documented mechanism for neuronal cell death in TSCI [46], and available data suggest that reduced extracellular Zn levels may exacerbate glutamate excitotoxicity in astrocytes and contribute to oligodendrocyte death [2]. Furthermore, Zn plays a crucial role in regulating Brain-Derived Neurotrophic Factor (BDNF), a key factor for neuroregeneration post-TSCI [47,48].

A key finding is the rapid increase in Zn and fZn levels in patients achieving AIS+2 outcomes within the first 24 h. This suggests that the ability to restore Zn homeostasis early post-injury can be critical for substantial neurological improvement. These patients not only showed higher baseline fZn but also exhibited greater resilience to prolonged Zn depletion, potentially reflecting better-preserved cellular integrity and reduced oxidative damage.

Our findings suggest that maintaining adequate Zn levels during the acute and subacute phases of TSCI could be critical for mitigating secondary injury processes, such as glutamate excitotoxicity and impaired neuroregeneration. Therefore, we hypothesize that therapeutic strategies aimed at stabilizing Zn homeostasis in the early post-injury 24 h period could reduce neuronal damage and enhance recovery by supporting BDNF-mediated neuroprotective mechanisms. Further studies are warranted to explore Zn supplementation or modulation as a potential therapeutic approach in TSCI patients.

#### 4.3.1. The Role of fZn in Immune Cell Signaling

Free Zn is a vital signaling molecule in immune cell communication, serving as a second messenger in various cellular processes and crucially regulating immune responses [8,26]. Zn influences numerous enzymes, transcription factors, and signaling pathways within immune cells, affecting T cells, B cells, and macrophages [4,5,7], which are integral to the adaptive and innate immune systems [10,22].

Zn’s role in modulating cytokine production further is essential for mediating and regulating immunity, inflammation, and hematopoiesis [5]. Zn deficiency disrupts immune cell development, activation, and maturation, leading to compromised host defense and increased inflammation risk [4]. It also impairs the functions of neutrophil granulocytes, including phagocytosis and oxidative burst, and affects monocytes and macrophages by modulating their phagocytic activity and cytokine secretion [5].

Zn’s role extends to natural killer (NK) cells, essential for the innate immune response against viral infections and tumors [49]. Adequate Zn levels enhance NK-cell numbers and activity, emphasizing its importance [49,50]. Maywald et al. (2017) further elucidate Zn’s regulatory functions, noting its critical involvement in intracellular signaling pathways and its impact on the immune system’s overall functionality [51]. Understanding fZn’s role in immune cell signaling is crucial for developing strategies to boost immune responses and improve health [33,51].

#### 4.3.2. TLR4/NF-κB/ZIP8 Pathway Activation and Zn Uptake in Monocytes

Further exploration of the molecular mechanisms underlying the decrease in serum Zn concentrations after TSCI is warranted. We speculate that the rapid changes may involve active uptake into monocytes, contributing to the observed alterations in peripheral serum Zn levels. The TSCI-induced activation of the TLR4/NF-κB/ZIP8 pathway is known to enhance Zn transport into monocytes [18], aligning with the broader understanding of immune-metabolic shifts in response to injury [52]. This pathway plays a pivotal role in regulating macrophage polarization [14,37,53], which diverges from the classical M1/M2 paradigm, instead reflecting a spectrum of dynamic and context-dependent states [52]. Promoting macrophage phenotypes that favor tissue repair and resolution of inflammation, previously described as M2-like but now viewed as part of a continuum, may yield therapeutic benefits [52]. Zn administration, shown to inhibit NF-κB signaling, aligns with this concept, attenuating excessive inflammation and promoting functional recovery in ischemic and TSCI models [6,20,53,54,55]. By shifting focus from rigid dichotomies to the nuanced understanding of immune cell plasticity, Zn homeostasis emerges as a potential therapeutic target. Modulating Zn uptake and redistribution in monocytes/macrophages offers a promising avenue for influencing neuroinflammatory responses and improving outcomes in TSCI patients.

#### 4.3.3. Mechanisms of Zn Homeostasis During Traumatic Spinal Cord Injury: Potential Role of Free Fatty Acids and Zn-Binding Proteins

It is particularly interesting to note that fZn levels exhibit less variation than total Zn levels. This raises two important questions: firstly, why does this occur? Is this a signaling mechanism or an attempt to maintain homeostasis? Secondly, how is the concentration of fZn regulated? While albumin is a key Zn-binding protein, it is not typically saturated with Zn under physiological conditions, suggesting other factors may be involved. The literature indicates that changes in Zn-binding proteins, such as albumin, during TSCI could influence Zn homeostasis [5,28,29]. Specifically, the interaction between free fatty acids (FFAs) and albumin can alter Zn binding capacity, as high levels of FFAs can displace Zn from albumin, redistributing it to other plasma proteins like histidine-rich glycoprotein (HRG) and components of the complement system, which are involved in immune responses [28,29]. This suggests that variations in FFA levels could play a significant role in maintaining stable fZn levels during the secondary injury phase after TSCI. Further research is required to explore these dynamics, potentially focusing on the impact of lipid metabolism changes in TSCI and their influence on Zn binding and availability.

### 4.4. Zn Dynamics in Relation to Injury Severity and Location

Patients with more severe injuries (AIS A or B at admission) exhibited lower baseline Zn levels and a slower recovery in both Zn and fZn concentrations over time. In contrast, those with less severe injuries (AIS C or D) showed faster normalization of Zn levels and a higher likelihood of achieving AIS+1 or AIS+2 outcomes. This suggests that initial injury severity modulates systemic Zn metabolism, potentially reflecting differential inflammatory responses and cellular repair mechanisms.

The significant drop in Zn within the first 4 h post-injury, particularly in AIS+2 patients, underscores the importance of Zn as a biomarker for early physiological stress. This hyperacute phase may represent a critical window where Zn depletion aligns with the activation of immune responses and oxidative stress pathways, which are more pronounced in patients showing substantial neurological recovery. The data suggest that patients with a stronger, more coordinated immune response may be more likely to experience meaningful improvements in AIS grades.

The observed differences in Zn dynamics between cervical and thoracic injuries further support the role of injury location in modulating Zn metabolism. Patients with thoracic injuries exhibited a more pronounced and sustained decline in Zn levels compared to those with cervical injuries. This aligns with previous findings suggesting that thoracic spinal cord damage often triggers more extensive systemic inflammation and autonomic dysregulation, potentially exacerbating Zn depletion.

The lack of significant change in fZn ratios across injury levels indicates that while total Zn fluctuates, the mechanisms regulating fZn remain relatively stable. This may reflect homeostatic processes aimed at preserving intracellular Zn availability, even during severe systemic depletion.

### 4.5. Clinical Implications

The clinical significance of these results is considerable. Firstly, monitoring Zn and free Zn levels could become a routine part of the assessment for patients with TSCI, providing crucial information about the potential severity of injury and guiding initial therapeutic decisions. Zn supplementation might be explored in future research as a therapeutic strategy to counteract the rapid loss of Zn following injury [55]. Furthermore, the distinct patterns observed in different injury levels (cervical versus thoracic) suggest that Zn supplementation protocols could be tailored based on the injury characteristics [11,18].

Secondly, the significant predictive value associated with Zn and free Zn measurements for neurological impairment could help in the prognostic assessment of patients. Identifying patients at high risk for severe neurological outcomes early in the treatment process can prioritize them for more aggressive management strategies, potentially improving outcomes [55]. Therefore, the current data can establish foundational serum Zn ranges for “more severe”, “less severe”, and control groups. These results should be analyzed using multicenter data to provide an initial approximation of clinically significant ranges when combined. Additionally, our study provides a promising model for early detection of patients at high risk of neurological impairment, which should be independently replicated.

Monitoring Zn and fZn levels could become a routine part of assessing patients with TSCI, offering crucial insights into injury severity and guiding therapeutic decisions. The possibility of using Zn supplements to counteract rapid depletion after injury warrants investigation, as promptly identifying high-risk patients could result in more specific and efficacious treatments, potentially enhancing patient recovery [55].

### 4.6. Limitations and Future Directions

Our explorative study offers substantial insights into the dynamics of serum and fZn concentrations in TSCI patients. Despite these advancements, several limitations suggest important directions for future research.

The relatively small sample size limits the generalizability of our findings across the broader TSCI population. The rarity and emergent nature of TSCI complicate extensive sample collection. Therefore, future studies would benefit from international collaborations that could pool larger, more diverse cohorts. This would not only provide more definitive evidence but also facilitate the development of clinical guidelines tailored to various TSCI severities and outcomes [56,57].

A significant challenge faced was the limited existing research on the physiological and pathological roles of fZn in general and especially after traumatic injuries, which substantially restricted the interpretability of our findings. While exclusion criteria reduce the trauma-related effects of injuries beyond SCI and column fractures, additional variation may arise from factors such as soft tissue injury. The lack of literature specifically addressing fZn homeostasis in critical health conditions highlights a critical gap in current scientific understanding [5,9]. To bridge this gap, future research must prioritize studies exploring the mechanisms of fZn homeostasis and its specific impacts on neurological recovery post-TSCI. This could involve detailed biochemical studies to map out the metabolic pathways influenced by fZn and its interactions with other trace elements and inflammatory markers [5,8,51].

A potential limitation of this study is the influence of diet on serum Zn levels and subsequent recovery, as dietary intake is a primary determinant of Zn availability. While the study measured Zn dynamics following traumatic spinal cord injury, it did not account for variations in patients’ dietary Zn intake prior to hospital admission. This limitation is partially mitigated by the relatively standardized nutritional regimens provided during intensive care and throughout hospital stays, which likely minimized variation in dietary Zn intake among participants after admission. However, differences in pre-hospital dietary habits may still have influenced baseline Zn levels and early post-injury fluctuations. Future studies should aim to incorporate dietary assessments or control for dietary intake to better isolate the impact of Zn dynamics on recovery outcomes.

Additionally, while our reliance on serum Zn as a biomarker provided valuable insights, it may not adequately reflect intracellular Zn status, which is more directly related to cellular function. This underscores the necessity of developing more precise biomarkers that can directly measure cellular Zn levels, particularly fZn, to improve diagnostic accuracy and therapeutic interventions [9].

The observational nature of this study also limits our ability to infer causation from the associations observed between Zn levels and neurological outcomes. Future mechanistic studies are needed to uncover the biological processes influenced by changes in Zn and fZn after TSCI, potentially leading to targeted therapies that modulate Zn levels to enhance neurological recovery [5,9,27].

Moreover, the neurological follow-up period in our study was limited to three months post-injury, which does not capture the full spectrum of recovery that can extend up to a year or longer. Future research should extend this timeline to better understand the long-term impacts of Zn dynamics on recovery and functional outcomes [58].

Variability due to demographic, and clinical factors not fully captured in our study might influence Zn levels, limiting the generalizability of our findings. Moreover, more efforts are needed to clarify the transferability of Zn homeostasis data across species, particularly as a surrogate parameter for the investigation of trauma-immunological impacts in TSCI.

While this study primarily focused on Zn and fZn levels, future investigations could benefit from incorporating intracellular Zn measurements by flow cytometry and single-cell RNA sequencing (scRNA-seq) to allow for the characterization of Zn distribution at the single-cell level, differentiating heterogeneity in immune responses following TSCI. These methods could identify Zn-responsive immune cell subpopulations, providing deeper insights into monocyte and macrophage polarization and their role in neuroinflammation [52].

Spatial imaging modalities such as laser ablation inductively coupled plasma mass spectrometry (LA-ICP-MS) and multiplex immunofluorescence could be employed to elucidate the spatial distribution of Zn within injured tissue, capturing local Zn dynamics at the site of neurotrauma. Applying these technologies in animal models would not only enable cross-species comparisons to validate Zn-based biomarkers in neurotrauma immunology but also offer translational potential for human TSCI studies. The integration of these approaches, combined with a more comprehensive understanding of demographic and clinical variability in larger multicenter cohort studies, could uncover novel biomarker signatures linked to Zn metabolism, informing targeted therapeutic strategies aimed at modulating immune responses and promoting neurological recovery for individuals who sustained TSCI.

## 5. Conclusions

This initial study on fZn in TSCI reveals early dynamics post-TSCI when compared to patients with without neurological impairment. The behavior of Zn and fZn highlights their potential roles in diagnostic and prognostic applications. The findings indicate a correlation between neurological impairments and swift reductions in serum Zn during the initial acute post-injury phase. Notably, the substantial alterations in fZn levels, detectable 24 h after injury, underline its utility in enhancing diagnostic precision, particularly in critically ill patients. The link between Zn dynamics and neurological remission further fuels the ongoing search for deeper understanding of how Zn affects recovery and function after TSCI. This understanding could pave the way for novel nutriceutical therapeutic approaches that focus on these particular pathways. Incorporating Zn level assessments as a covariate in biomarker signature analysis for TSCI clinical management might improve early diagnosis and patient care for individuals with TSCI.

## Figures and Tables

**Table 1:** Descriptive statistics for individuals with traumatic spinal cord injury (TSCI), categorized by initial American Spinal Injury Association Impairment Scale (AIS) grade and remission status. AIS+0 indicates no improvement, AIS+1 represents a one-grade improvement, and AIS+2 reflects a two-grade improvement. Baseline characteristics such as sex, age, AO classification, and neurological level of injury (NLI) are presented.

Variables	AIS+:0 (N = 21)	AIS+: ≥1 (N = 18)	Total (N = 39)	*p*-Value
**Sex**				0.139 ($\chi^{2}$)
M	19 (90%)	13 (72%)	32 (82%)	
F	2 (10%)	5 (28%)	7 (18%)	
**Age**				0.183 (*t*-test)
N	21	18	39	
Mean	57	49	54	
SD	20	18	19	
Median	60	52	53	
Q1–Q3	41–76	34–59	36–67	
Min–Max	22–85	20–79	20–85	
**AO Classification**				0.186 ($\chi^{2}$)
A	8 (38%)	12 (67%)	20 (51%)	
B	3 (14%)	2 (11%)	5 (13%)	
C	10 (48%)	4 (22%)	14 (36%)	
**NLI**				0.213 ($\chi^{2}$)
Thoracical	11 (52%)	5 (28%)	16 (41%)	
Lumbar	1 (5%)	3 (17%)	4 (10%)	
Cervical	9 (43%)	10 (56%)	19 (49%)	

**Table 2:** Descriptive statistics comparing the control group patients who have vertebral fractures without any neurological deficits (C) and study group (S).

Variables	Control (C, N = 9)	Study (S, N = 39)	*p*-Value
**Sex**			0.019 ($\chi^{2}$)
F	5 (56%)	7 (18%)	
M	4 (44%)	32 (82%)	
**Age**			0.224 (*t*-test)
N	9	39	
Mean	46	54	
SD	16	19	
Median	40	53	
Q1–Q3	32–59	36–67	
Min–Max	27–71	20–85	
**AO Classification**			0.067 ($\chi^{2}$)
A	6 (67%)	20 (51%)	
B	3 (33%)	5 (13%)	
C	0 (0%)	14 (36%)	

**Table 3:** All continuous predictors are mean-centered and scaled by 1 standard deviation. The outcome variable is in its original units. *** p<0.001; ** p<0.01; * p<0.05.

	Model 1	Model 2	Model 3	Cohen’s d
(Intercept)	1.64 ** [0.64, 2.63]	1.79 *** [0.74, 2.83]	1.48 *** [0.64, 2.33]	
Zn 9 h	−1.02 * [−1.97, −0.06]			0.90 large
fZn 24 h		−1.54 ** [−2.67, −0.40]		1.33 large
Zn/fZn 1 d			−0.89 * [−1.76, −0.02]	0.82 large
N	35	42	42	
AIC	33.61	36.25	42.66	
BIC	36.72	39.72	46.14	
Pseudo R^2^	0.23	0.37	0.17	

## Data Availability

Data supporting the findings of this study are available from the corresponding author upon reasonable request.

## Abbreviations

The following abbreviations are used in this manuscript:

AIS	American Spinal Injury Association Impairment Scale
AUC	Area Under the Curve
BDNF	Brain-Derived Neurotrophic Factor
CI	Confidence Interval
CNS	Central Nervous System
FFAs	Free Fatty Acids
fZn	Free Zinc
HRG	Histidine-Rich Glycoprotein
NF-κB	Nuclear Factor Kappa B
NLI	Neurological Level of Injury
TLR4	Toll-Like Receptor 4
TSCI	Traumatic Spinal Cord Injury
Zn	Zinc

## References

[1] Ackery A., Tator C., Krassioukov A. (2004). A global perspective on spinal cord injury epidemiology. J. Neurotrauma.

[2] Alizadeh A., Dyck S.M., Karimi-Abdolrezaee S. (2019). Traumatic Spinal Cord Injury: An Overview of Pathophysiology, Models and Acute Injury Mechanisms. Front. Neurol..

[3] Ahuja C.S., Badhiwala J.H., Fehlings M.G. (2020). “Time is spine”: The importance of early intervention for traumatic spinal cord injury. Spinal Cord.

[4] Gammoh N.Z., Rink L. (2017). Zinc in Infection and Inflammation. Nutrients.

[5] Maares M., Haase H. (2016). Zinc and immunity: An essential interrelation. Arch. Biochem. Biophys..

[6] Kijima K., Ono G., Kobayakawa K., Saiwai H., Hara M., Yoshizaki S., Yokota K., Saito T., Tamaru T., Iura H. (2023). Zinc deficiency impairs axonal regeneration and functional recovery after spinal cord injury by modulating macrophage polarization via NF-kappaB pathway. Front. Immunol..

[7] Fukada T., Yamasaki S., Nishida K., Murakami M., Hirano T. (2011). Zinc homeostasis and signaling in health and diseases: Zinc signaling. J. Biol. Inorg. Chem..

[8] Maares M., Hackler J., Haupt A., Heller R.A., Bachmann M., Diegmann J., Moghaddam A., Schomburg L., Haase H. (2022). Free Zinc as a Predictive Marker for COVID-19 Mortality Risk. Nutrients.

[9] Hoeger J., Simon T.P., Doemming S., Thiele C., Marx G., Schuerholz T., Haase H. (2015). Alterations in zinc binding capacity, free zinc levels and total serum zinc in a porcine model of sepsis. Biometals.

[10] Wessels I., Maywald M., Rink L. (2017). Zinc as a Gatekeeper of Immune Function. Nutrients.

[11] Heller R.A., Sperl A., Seelig J., Haubruck P., Bock T., Werner T., Besseling A., Sun Q., Schomburg L., Moghaddam A. (2020). Zinc Concentration Dynamics Indicate Neurological Impairment Odds after Traumatic Spinal Cord Injury. Antioxidants.

[12] Rink L., Gabriel P. (2000). Zinc and the immune system. Proc. Nutr. Soc..

[13] Wessels I., Pupke J.T., von Trotha K.T., Gombert A., Himmelsbach A., Fischer H.J., Jacobs M.J., Rink L., Grommes J. (2020). Zinc supplementation ameliorates lung injury by reducing neutrophil recruitment and activity. Thorax.

[14] Haase H., Ober-Blobaum J.L., Engelhardt G., Hebel S., Heit A., Heine H., Rink L. (2008). Zinc signals are essential for lipopolysaccharide-induced signal transduction in monocytes. J. Immunol..

[15] Haase H., Hebel S., Engelhardt G., Rink L. (2006). Flow cytometric measurement of labile zinc in peripheral blood mononuclear cells. Anal. Biochem..

[16] Hu H., Xia N., Lin J., Li D., Zhang C., Ge M., Tian H., Mei X. (2021). Zinc Regulates Glucose Metabolism of the Spinal Cord and Neurons and Promotes Functional Recovery after Spinal Cord Injury through the AMPK Signaling Pathway. Oxid. Med. Cell Longev..

[17] Garcia E., Hernandez-Ayvar F., Rodriguez-Barrera R., Flores-Romero A., Borlongan C., Ibarra A. (2022). Supplementation With Vitamin E, Zinc, Selenium, and Copper Re-Establishes T-Cell Function and Improves Motor Recovery in a Rat Model of Spinal Cord Injury. Cell Transpl..

[18] Kijima K., Kubota K., Hara M., Kobayakawa K., Yokota K., Saito T., Yoshizaki S., Maeda T., Konno D., Matsumoto Y. (2019). The acute phase serum zinc concentration is a reliable biomarker for predicting the functional outcome after spinal cord injury. EBioMedicine.

[19] Li D., Tian H., Li X., Mao L., Zhao X., Lin J., Lin S., Xu C., Liu Y., Guo Y. (2020). Zinc promotes functional recovery after spinal cord injury by activating Nrf2/HO-1 defense pathway and inhibiting inflammation of NLRP3 in nerve cells. Life Sci..

[20] Lin J.Q., Tian H., Zhao X.G., Lin S., Li D.Y., Liu Y.Y., Xu C., Mei X.F. (2021). Zinc provides neuroprotection by regulating NLRP3 inflammasome through autophagy and ubiquitination in a spinal contusion injury model. CNS Neurosci. Ther..

[21] Haruta Y., Kobayakawa K., Saiwai H., Hata K., Tamaru T., Iura H., Ono G., Kitade K., Kijima K., Iida K. (2022). Zinc chelator treatment in crush syndrome model mice attenuates ischemia-reperfusion-induced muscle injury due to suppressing of neutrophil infiltration. Sci. Rep..

[22] Wu D., Lewis E.D., Pae M., Meydani S.N. (2018). Nutritional Modulation of Immune Function: Analysis of Evidence, Mechanisms, and Clinical Relevance. Front. Immunol..

[23] Xu C., Zhou Z., Zhao H., Lin S., Zhang P., Tian H., Mei X. (2023). Zinc Promotes Spinal Cord Injury Recovery by Blocking the Activation of NLRP3 Inflammasome Through SIRT3-Mediated Autophagy. Neurochem. Res..

[24] Paterniti I., Filippone A., Naletova I., Greco V., Sciuto S., Esposito E., Cuzzocrea S., Rizzarelli E. (2023). Trehalose-Carnosine Prevents the Effects of Spinal Cord Injury Through Regulating Acute Inflammation and Zinc(II) Ion Homeostasis. Cell Mol. Neurobiol..

[25] Besecker B.Y., Exline M.C., Hollyfield J., Phillips G., Disilvestro R.A., Wewers M.D., Knoell D.L. (2011). A comparison of zinc metabolism, inflammation, and disease severity in critically ill infected and noninfected adults early after intensive care unit admission. Am. J. Clin. Nutr..

[26] Maywald M., Wang F., Rink L. (2018). The Intracellular Free Zinc Level Is Vital for Treg Function and a Feasible Tool to Discriminate between Treg and Activated Th Cells. Int. J. Mol. Sci..

[27] Alker W., Schwerdtle T., Schomburg L., Haase H. (2019). A Zinpyr-1-based Fluorimetric Microassay for Free Zinc in Human Serum. Int. J. Mol. Sci..

[28] Coverdale J.P.C., Barnett J.P., Adamu A.H., Griffiths E.J., Stewart A.J., Blindauer C.A. (2019). A metalloproteomic analysis of interactions between plasma proteins and zinc: Elevated fatty acid levels affect zinc distribution. Metallomics.

[29] Coverdale J.P.C., Khazaipoul S., Arya S., Stewart A.J., Blindauer C.A. (2019). Crosstalk between zinc and free fatty acids in plasma. Biochim. Biophys. Acta Mol. Cell Biol. Lipids.

[30] Doering P., Stoltenberg M., Penkowa M., Rungby J., Larsen A., Danscher G. (2010). Chemical blocking of zinc ions in CNS increases neuronal damage following traumatic brain injury (TBI) in mice. PLoS ONE.

[31] Olson L.M., Coffey R., Porter K., Thomas S., Bailey J.K., Jones L.M., Murphy C.V. (2020). The impact of serum zinc normalization on clinical outcomes in severe burn patients. Burns.

[32] Han M., Lin W.H., Huang S.H., Lin Z.X., Li K.S. (2023). Association between plasma metal elements and platelet dysfunction in trauma-induced coagulopathy rat model. J. Trace Elem. Med. Biol..

[33] Huang S.Y., Huang J.F., Chan S.Y., Yang C.H.O., Cheng C.T., Wang C.C., Hsu C.P., Fu C.Y., Liao C.H. (2023). Effect of zinc supplement on patients with trauma: A systematic review and meta-analysis. J. Parenter. Enter. Nutr..

[34] World Medical Association (2013). World Medical Association Declaration of Helsinki: Ethical principles for medical research involving human subjects. JAMA.

[35] Collins G.S., Reitsma J.B., Altman D.G., Moons K.G. (2015). Transparent reporting of a multivariable prediction model for individual prognosis or diagnosis (TRIPOD): The TRIPOD statement. BMJ.

[36] Bock T., Heller R.A., Haubruck P., Raven T.F., Pilz M., Moghaddam A., Biglari B. (2021). Pursuing More Aggressive Timelines in the Surgical Treatment of Traumatic Spinal Cord Injury (TSCI): A Retrospective Cohort Study with Subgroup Analysis. J. Clin. Med..

[37] Heller R.A., Seelig J., Crowell H.L., Pilz M., Haubruck P., Sun Q., Schomburg L., Daniel V., Moghaddam A., Biglari B. (2021). Predicting neurological recovery after traumatic spinal cord injury by time-resolved analysis of monocyte subsets. Brain.

[38] Magerl F., Aebi M., Gertzbein S.D., Harms J., Nazarian S. (1994). A comprehensive classification of thoracic and lumbar injuries. Eur. Spine J..

[39] Boschloo R.D. (1970). Raised conditional level of significance for the 2 × 2-table when testing the equality of two probabilities. Stat. Neerl..

[40] R Core Team (2024). R: A Language and Environment for Statistical Computing.

[41] Wickham H., Chang W., Wickham M.H. (2016). Package ‘ggplot2’. Creat. Elegant Data Vis. Using Gramm. Graphics. Version.

[42] Jafari A., Noormohammadi Z., Askari M., Daneshzad E. (2022). Zinc supplementation and immune factors in adults: A systematic review and meta-analysis of randomized clinical trials. Crit. Rev. Food Sci. Nutr..

[43] Hosseini R., Ferns G.A., Sahebkar A., Mirshekar M.A., Jalali M. (2021). Zinc supplementation is associated with a reduction in serum markers of inflammation and oxidative stress in adults: A systematic review and meta-analysis of randomized controlled trials. Cytokine.

[44] Mousavi S.M., Djafarian K., Mojtahed A., Varkaneh H.K., Shab-Bidar S. (2018). The effect of zinc supplementation on plasma C-reactive protein concentrations: A systematic review and meta-analysis of randomized controlled trials. Eur. J. Pharmacol..

[45] Ahuja C.S., Nori S., Tetreault L., Wilson J., Kwon B., Harrop J., Choi D., Fehlings M.G. (2017). Traumatic Spinal Cord Injury-Repair and Regeneration. Neurosurgery.

[46] Takeda A. (2011). Insight into Glutamate Excitotoxicity from Synaptic Zinc Homeostasis. Int. J. Alzheimer’s Dis..

[47] Garraway S.M., Huie J.R. (2016). Spinal Plasticity and Behavior: BDNF-Induced Neuromodulation in Uninjured and Injured Spinal Cord. Neural Plast..

[48] Ritfeld G.J., Patel A., Chou A., Novosat T.L., Castillo D.G., Roos R.A.C., Oudega M. (2015). The Role of Brain-Derived Neurotrophic Factor in Bone Marrow Stromal Cell-Mediated Spinal Cord Repair. Cell Transplant..

[49] Mocchegiani E., Giacconi R., Cipriano C., Malavolta M. (2009). NK and NKT cells in aging and longevity: Role of zinc and metallothioneins. J. Clin. Immunol..

[50] Fraker P.J., King L.E., Laakko T., Vollmer T.L. (2000). The dynamic link between the integrity of the immune system and zinc status. J. Nutr..

[51] Maywald M., Wessels I., Rink L. (2017). Zinc Signals and Immunity. Int. J. Mol. Sci..

[52] Paolicelli R.C., Sierra A., Stevens B., Tremblay M.E., Aguzzi A., Ajami B., Amit I., Audinat E., Bechmann I., Bennett M. (2022). Microglia states and nomenclature: A field at its crossroads. Neuron.

[53] Shechter R., Miller O., Yovel G., Rosenzweig N., London A., Ruckh J., Kim K.W., Klein E., Kalchenko V., Bendel P. (2013). Recruitment of beneficial M2 macrophages to injured spinal cord is orchestrated by remote brain choroid plexus. Immunity.

[54] Shechter R., London A., Varol C., Raposo C., Cusimano M., Yovel G., Rolls A., Mack M., Pluchino S., Martino G. (2009). Infiltrating blood-derived macrophages are vital cells playing an anti-inflammatory role in recovery from spinal cord injury in mice. PLoS Med.

[55] Aguilar-Peralta A.K., Gonzalez-Vazquez A., Tomas-Sanchez C., Blanco-Alvarez V.M., Martinez-Fong D., Gonzalez-Barrios J.A., Limon I.D., Millán-Perez Peña L., Flores G., Soto-Rodriguez G. (2022). Prophylactic Zinc Administration Combined with Swimming Exercise Prevents Cognitive-Emotional Disturbances and Tissue Injury following a Transient Hypoxic-Ischemic Insult in the Rat. Behav. Neurol..

[56] Hoekstra F., Trigo F., Sibley K.M., Graham I.D., Kennefick M., Mrklas K.J., Nguyen T., Vis-Dunbar M., Gainforth H.L. (2023). Systematic overviews of partnership principles and strategies identified from health research about spinal cord injury and related health conditions: A scoping review. J. Spinal Cord Med..

[57] Noonan V.K., Chan E., Bassett-Spiers K., Berlowitz D.J., Biering-Sørensen F., Charlifue S., Graco M., Hayes K.C., Horsewell J., Joshi P. (2017). Facilitators and Barriers to International Collaboration in Spinal Cord Injury: Results from a Survey of Clinicians and Researchers. J. Neurotrauma.

[58] Kirshblum S., Snider B., Eren F., Guest J. (2020). Characterizing Natural Recovery after Traumatic Spinal Cord Injury. J. Neurotrauma.

